# Effectiveness of transcutaneous auricular vagus nerve stimulation in stroke rehabilitation: a systematic review and meta-analysis of randomized clinical trials

**DOI:** 10.3389/fneur.2026.1786103

**Published:** 2026-05-04

**Authors:** Shuaijing Wan, Xiaolu Liu, Wenjing Jiang, Zesen Li, Zhexuan Yan, Yu Yin, Weibo Li

**Affiliations:** 1 College of Nursing and Rehabilitation, North China University of Science and Technology, Tangshan, China; 2 Department of Rehabilitation, Hebei General Hospital, Shijiazhuang, China; 3 Hebei Provincial Key Laboratory of Cerebral Networks and Cognitive Disorders, Shijiazhuang, China; 4 Graduate School of Hebei Medical University, Shijiazhuang, China; 5 Physical Education College, Hebei Normal University, Shijiazhuang, China; 6 Department of Gastrointestinal Surgery, The Second hospital of Hebei Medical University, Shijiazhuang, China

**Keywords:** meta-analysis, motor, stroke, taVNS, transcutaneous auricular vagus nerve stimulation

## Abstract

**Background and aim:**

Transcutaneous auricular vagus nerve stimulation (taVNS) has demonstrated potential efficacy in post-stroke functional recovery. This study aimed to systematically synthesize data evaluating the effects of taVNS in terms of improving motor function, mental health, and activities of daily living (ADL) in patients experiencing stroke following intervention.

**Methods:**

Electronic databases including EMBASE, Cochrane Library, PubMed, Web of Science, China National Knowledge Infrastructure, Wanfang, and VIP were searched from their inception to September 2025. All randomized controlled trials that applied taVNS to patients experiencing stroke were included.

**Results:**

Ten randomized controlled trials (RCTs) involving 512 patients were included in the analysis. The results showed that compared with the control group, the taVNS group demonstrated significantly increased motor function scores [standardized mean difference (SMD) = 1.21; 95% CI: 0.88–1.55; *p* < 0.001], significantly improved mental health scores (SMD = 0.84, 95% CI: 1.19,–0.49; *p* < 0.001), significantly increased scores in ADL (SMD = 0.94; 95% CI: 0.72–1.17; *p* < 0.001), and significantly different neurophysiological indicators (SMD = 1.60, 95% CI: 0.70–2.51; *p* = 0.0005). Subgroup analysis revealed superior outcomes in patients with stroke who received 20 Hz taVNS with ≥10 sessions.

**Conclusion:**

taVNS improves motor function, mental health, and ADL outcomes in patients experiencing stroke. The combination of taVNS stimulation frequency, current intensity, and intervention duration constitutes a key modulator influencing treatment efficacy.

**Systematic review registration:**

https://www.crd.york.ac.uk/PROSPERO/view/CRD42025633212, identifier PROSPERO (CRD42025633212).

## Highlights

- In the previous meta-analysis on the effect of taVNS on function in stroke patients, only the study protocols of the trials were analyzed.
- This study builds on that by retrieving and conducting an in-depth analysis of published articles.
- The study focuses on diverse functional dimensions, including motor, mental health, ADL, and swallowing functions.
- Although multiple functional dimensions are considered, the sample size of the study is relatively small.

## Introduction

Stroke remains a leading cause of long-term disability worldwide, often resulting in persistent motor impairments, mental health issues, and reduced ability to perform activities of daily living (ADL), thereby significantly diminishing the quality of life of patients (1, 2). Despite continuous advancements in traditional rehabilitation therapies, these approaches remain inadequate in promoting full functional recovery, particularly in the chronic phase of stroke (3). However, preclinical studies indicate that animals in the chronic phase of stroke may still regain motor function through motor network reorganization and cortical plasticity (4). This indicates an urgent need to develop novel, effective therapies integrated with rehabilitation treatment to maximize neural plasticity and amplify the benefits of rehabilitation therapy.

Vagus nerve stimulation (VNS) has emerged as a promising modality for promoting recovery after stroke (5). Traditional invasive VNS requires surgical implantation of devices. Both preclinical studies and clinical trials indicate that combining traditional invasive VNS with rehabilitation training can effectively improve upper limb function (6, 7). However, its invasive nature, associated costs, and potential risks have limited the clinical adoption of this technology (8). Transcutaneous auricular VNS (taVNS), a noninvasive technique that stimulates the auricular branch of the vagus nerve, has garnered significant attention in recent years (9). As a safe, accessible, and well-tolerated alternative, taVNS offers a new pathway to harness the neuroplasticity potential of VNS without surgical intervention.

An increasing number of randomized controlled trials (RCTs) have started exploring the efficacy of taVNS combined with rehabilitation therapy on various functional outcomes in patients experiencing stroke (3, 10). However, the research findings are inconsistent, and the available findings exhibit significant heterogeneity in terms of stimulation parameters, patient populations (e.g., acute, subacute, or chronic stroke), and outcome measures. Systematic reviews have examined the overall effects of VNS (10). However, few studies have specifically focused on emerging evidence for this percutaneous stimulation modality. Furthermore, existing systematic reviews predominantly focus on a single functional impairment, failing to comprehensively integrate the latest RCT evidence to evaluate efficacy across multiple domains of stroke recovery or examine the effects of different stimulation protocols.

Therefore, this systematic review and meta-analysis aimed to determine the overall effectiveness of taVNS on motor function, mental health, ADL, and neurophysiological dimensions in patients experiencing stroke; identify factors influencing its efficacy, and guide clinical practice while advancing future research in this field.

## Methods

### Search strategy and selection criteria

This meta-analysis was conducted and reported as per the Preferred Reporting Items for Systematic Reviews and MetaAnalyses guidelines (11). This systematic review was registered with PROSPERO (CRD42025633212).

We systematically searched four English electronic databases (EMBASE, Cochrane Library, PubMed, and Web of Science) and three Chinese electronic databases (China National Knowledge Infrastructure, Wanfang Data, and VIP), covering the period from their inception to September 2025. The aim was to identify RCTs comparing the effects of “transcutaneous auricular VNS” versus “any control group” on functional recovery in patients experiencing stroke. We also searched major trial registries, including ClinicalTrials.gov, the World Health Organization Clinical Trials Registry Platform, and the China Clinical Trials Registry. The search terms included “stroke” and “transcutaneous auricular VNS.”

This review included original research articles examining taVNS for function recovery in patients with stroke. The eligibility criteria (based on the PICOS framework) (12) were as follows: (1) P: The target population included participants aged more than 18 years with a clinical diagnosis of a first stroke. (2) I: The intervention groups received active taVNS therapy. (3) C: The control groups received sham taVNS therapy or no taVNS therapy. (4) O: The main efficacy outcome was motor function assessed with the Fugl–Meyer Assessment (FMA) test, Wolf Motor Function Test (WFMT), action research arm test (ARAT), timed up and go test (TUG), and Berg Balance Scale (BBS) at the end of follow-up. The additional efficacy outcomes were mental health examined using the 17-Item Hamilton Depression Rating Scale (HAMD-17), Self-Rating Depression Scale (SDS), Hospital Anxiety Depression Scale-Anxiety Scale (HADS-A) or [HADS-Depression Scale (HADS-D)], ADL measured with Barthel Index (BI), modified Barthel Index (MBI) or Functional Independence Measure (FIM), and neuroelectrophysiological parameters such as motor evoked potential (MEP) amplitude and surface electromyography (sEMG). (5) S: We included peer-reviewed RCTs with a minimum of 10 participants published in English reporting results for taVNS therapy paired/combined with rehabilitation in patients who had a stroke.

The exclusion criteria were as follows: (1) non-RCT design; (2) incomplete outcome data; and (3) composite interventions.

### Identification of relevant studies and data extraction

Two researchers (SJW and WJJ) independently screened the retrieved trials for eligibility based on the aforementioned inclusion and exclusion criteria. Full texts were assessed, and data, including participant characters, taVNS parameters, intervention designs, and outcome measures, were extracted from the included articles. If the data were missing or incomplete, the corresponding authors were contacted. Any disagreements for study inclusion were resolved by consensus with a third reviewer (ZXY).

### Quality assessment

Two reviewers independently assessed the methodological quality of the included trials with the Physiotherapy Evidence Database (PEDro) Scale (13). Trials with the PEDro Scale scored six or higher were considered high quality. The Version 2 of Cochrane revised risk-of-bias tool for randomized trials (RoB 2) was used to assess risk of bias of the included studies (14). The potential bias on six domains, including randomization process, deviations from intended interventions, missing outcome data, measurement of the outcome, selection of the reported result, and overall bias, was determined. We judged the overall risk of bias of the included trials as low, high, or some concerns (14). Any disagreements were resolved by consultation with a third investigator.

### Evaluation of evidence level

We will use the GRADE approach to assess the certainty of the evidence and the strength of recommendations (15). The GRADEpro GDT software^1^ will be used to generate concise ‘Summary of Findings’ tables for our key outcomes:

Primary outcomes: motor function.

Secondary outcomes: Mental health, ADL and neuroelectrophysiological

Two reviewers will independently rate the certainty of the evidence across the five GRADE domains, namely risk of bias, inconsistency, indirectness, imprecision and publication bias, and assign overall grades of high, moderate, low or very low.

### Statistical analysis

The RevMan 5.4 software was used to perform the meta-analysis. Changes in values before and after treatment were analyzed and expressed as mean ± standard deviation. The mean difference analysis was used for continuous data selected from the same scale. Standardized mean difference (SMD) analyses were used for outcome indicators with different measurement scales (16). Heterogeneity was quantified using the *I* (2) statistic, and the statistical heterogeneity was interpreted as follows: low, ≤25%; moderate, >25% and ≤75%; or high, >75%. A random-effects model was used to calculate the combined effect sizes and 95% confidence intervals (17). Subgroup analyses for combined efficacy outcomes of taVNS post-intervention were performed to identify potential factors for rehabilitation efficacy. The analyzed subgroup types included the following: (1) phase of stroke onset [acute phase (<14 days), subacute phase (15 days to 6 months), and chronic phase (>6 months)]; (2) taVNS frequency (20, 25, and 30 Hz); (3) taVNS pulse width (200, 300, and 500 ms); (4) total intervention course (≤10 sessions, 11–20 sessions, and >20 sessions); and (5) weekly frequency of taVNS (≤4 sessions and >4 sessions).

## Results

### Literature review

**Figure 1:**
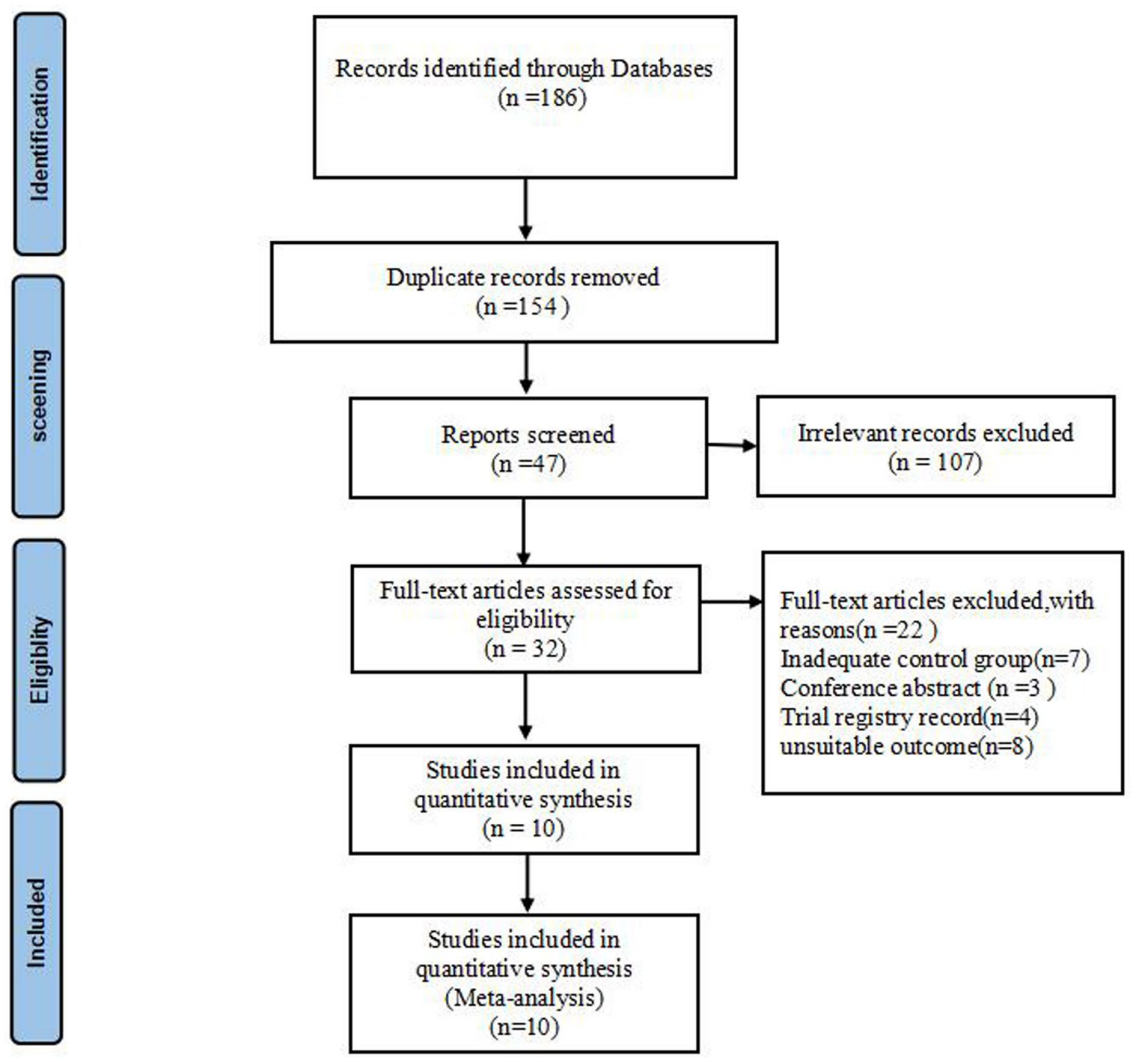
PRISMA flowchart showing the screening process.

The report screening process for this review is illustrated in Figure 1, with 10 RCTs included. The summary of patient characteristics and methodological features of all included trials is presented in Table 1.

**Table 1:** Overview of patient characters and methodological features in systematic review.

Study	Patients characteristics							taVNS stimulation parameters						Outcome measure	PEDro scale
	Size (*n*)	Age (year)	Sex (M/F)	Phase of stroke onset	Disease duration (mean ± SD)	Type (H/I)	Dysfunction type	Stimulator placement	Amplitude (mA)	Frequency (Hz)	Pulse width (μs)	Pulses per session/ per week/session	Treatment order		
Badran et al. (8)	T: 9	57.33 ± 8.23	4/5	Chronic	3.22 ± 3.14 years	NR	Motor	Left and right ears Cymba conchae and tragus	1.43 ± 0.17	25	500	45,000/4/12	Precise pairing	FMA-UEWMFT	8
	C: 7	58.71 ± 6.45	5/5		4.51 ± 3.93 years								Unpaired		
Liu et al. (39)	T: 40	62.60 ± 9.20	22/18	Acute/subacute	17.30 ± 7.10 days	35/5	Depression	Left auricular cavum conchae	1.82 ± 0.4	20	300	5400/4/28	NR	HAMD SDS BI	10
	C: 40	64.30 ± 10.10	25/15		18.40 ± 6.80 days	33/7									
Wang et al. (19)	T: 44	60.82 ± 6.19	21/23	Subacute	19.24 ± 5.83 days	20/24	Motor (gait)	Tragus of the left ear	NR	25	300	22,500/4/20	taVNS first	FMA-LE MBI BBS	7
	C: 40	61.94 ± 3.28	17/23		18.89 ± 5.17 days	24/16									
Capone et al. (34)	T: 7	53.71 ± 5.88	4/3	Chronic	93.71 ± 38.81 months	2/5	Motor	Left external acoustic meatus at the inner side of the tragus	2.0–4.5	20	300	600/2/10	taVNS first	FMA-UE	8
	C: 5	55.60 ± 7.12	3/2		46.00 ± 21.85 months	2/3									
Chang et al. (40)	T: 18	59.02 ± 1.98	18/18	Chronic	2.16 ± 0.39 years	9/7	Motor	Cymba conchae region of the left ear	0.1–5.0	30	300	3840/3/9	taVNS first	FMA-UE sEMG	8
	C: 18														
Li et al. (18)	T: 30	62.20 ± 12.30	15/15	Acute/subacute	10.80 ± 7.70 days	3/27	Motor Sensory and emotional	Left auricular cavum conchae	1.71 ± 0.5	20	300	2400/4/20	taVNS first	FMA-UE FMA-LEWMFT HAMD	10
	C: 30	68.30 ± 12.10	14/16		10.40 ± 6.90 days	2/28									
Wang et al. (20)	T: 20	55.00 ± 11.00	2/18	Subacute	3.20 ± 2.04 months	7/13	Motor	Left auricular cymba concha	6.55 ± 1.57	25	500	45,000/4/20	Simultaneously	FMA-UE ARAT MEP	10
	C: 20	57.00 ± 11.00	5/15		4.15 ± 1.60 months	6/14									
Zhang et al. (41)	T: 63	62.67 ± 7.34	38/25	Subacute	4.28 ± 1.46 months	NR	Motor	Left ear canal	NR	4–20	200	NR/4/24	Simultaneously	FMA-UEMBI MEP	6
	C: 61	61.59 ± 7.87	36/25		4.22 ± 1.35 months										
Wang et al. (26)	T: 19	60.26 ± 5.65	9/10	Acute/subacute	22.00 ± 3.34 days	4/15	Swallowing	Left and right ears	1.83 ± 0.5	25	500	NR/3/30	NR	MASA FCM RAS	7
	C: 20	58.25 ± 6.86	8/12		23.70 ± 2.95 days	6/14									
Wu et al. (3)	T: 10	64.50 ± 9.97	5/5	Subacute	36.30 ± 9.23 days	0/10	Motor	Left cymba conchae	1.66 ± 0.4	20	300	600/3/15	taVNS first	FMA-UEWMFT	7
	C: 11	61.82 ± 10.63	8/3		35.55 ± 6.47 days	0/11									

ARAT, action research arm test; BBS, Berg Balance Scale; BI, Barthel Index; C, control group; FCM, functional communication measure swallowing test; FMA-LE, Fugl–Meyer Assessment of Lower Extremity; FMA-UE, Fugl–Meyer Assessment of Upper Extremity; HAMD, Hamilton Depression Rating Scale; HADS, Hospital Anxiety Depression Scale; MASA, Mann Assessment of Swallowing Ability; MBI, modified Barthel Index; MEP, motor evoked potential; NR, not reported; RAS, Rosenbek aspiration scale; SDS, Self-Rating Depression Scale; sEMG, surface electromyography; T, treatment group; WMFT, Wolf Motor Function Test.

All trials employed RCT designs, targeting post-stroke functional impairments including motor, sensory, cognitive, and swallowing dysfunction. However, only one trial investigated the effects of taVNS on swallowing function, and therefore data from this study were included solely in the pooled analysis. No separate analysis was conducted to assess the impact of taVNS on post-stroke dysphagia. The data from WFMT in one study (18) were not included in this meta-analysis because the original paper reported only baseline data and no post-intervention data. Also, we could not obtain these data from the corresponding author or estimate them from figures. Additionally, the baseline and post-intervention data from six studies were converted into mean change values and standard deviations for data analysis. Ultimately, the data from 10 studies (total sample size of 512 participants) were included in the meta-analysis.

**Figure 2:**
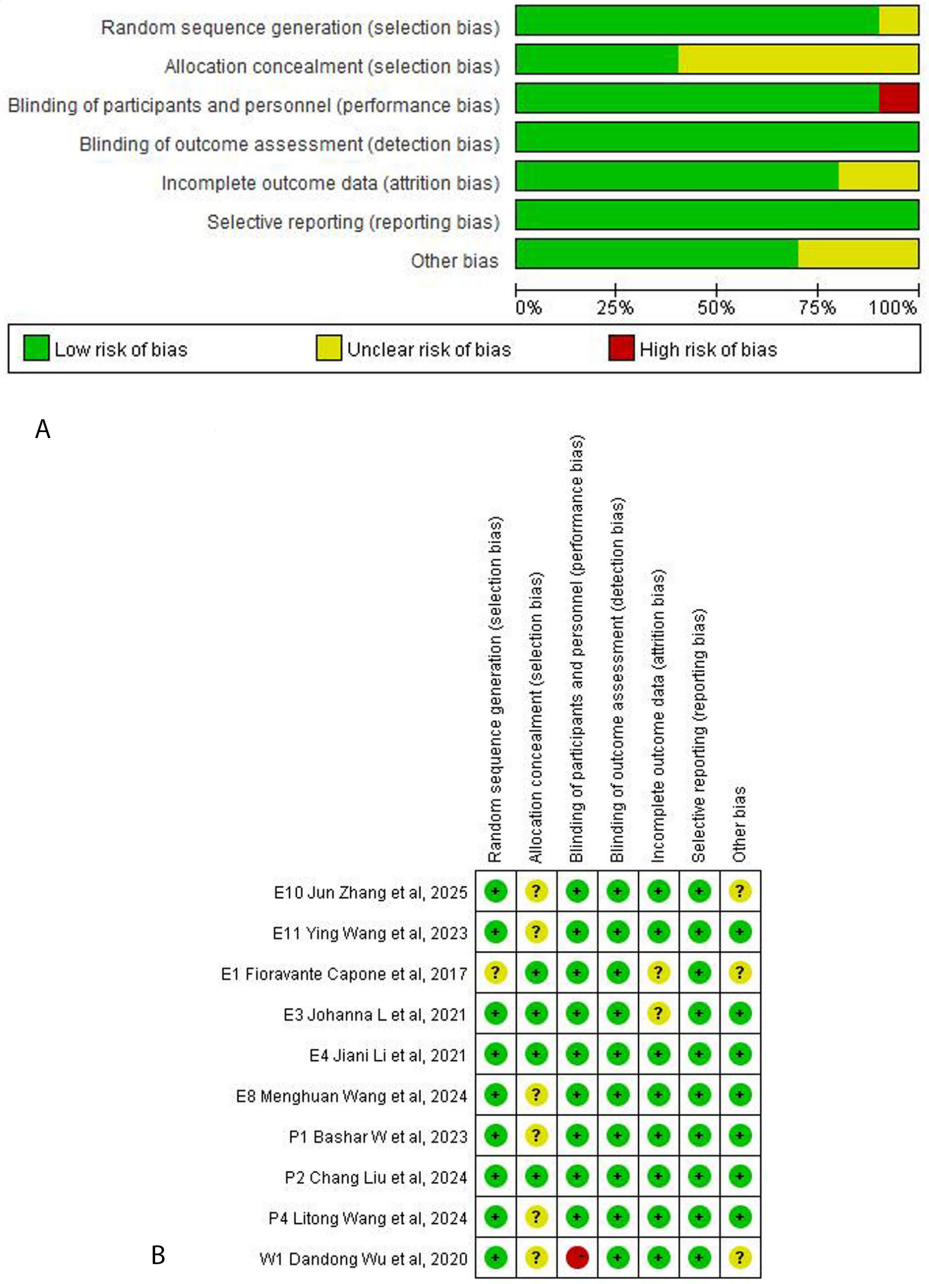
Inclusion of the risk-of-bias graph from this meta-analysis. **(A)** Risk-of-bias graph. **(B)** Risk-of-bias summary.

Our quantitative analysis using the PEDro Scale indicated that all RCTs included in the literature review were of high quality (Table 1). The assessment of RoB 2 revealed that the included studies exhibited varying levels of risk of bias: two studies had a low overall risk of bias, whereas eight studies had a moderate risk of bias, as shown in Figure 2. Summary of findings tables systematically present numerical results that show the impact of alternative interventions on prioritised patient important outcomes, and the associated certainty of evidence, as shown in Table 2.

**Table 2:** Summary of findings table.

Outcome	Study design	Risk of bias	Inconsistency	Indirectness	Imprecision	Absolute effect estimates (95% CI)	Certainty of evidence	Plain language summary
Motor	Randomised trials	Not serious	Serious^a^	Serious^b^	Not serious	SMD 1.13 higher (0.85 higher to 1.41 higher)	⨁⨁⨁◯ Moderate^a^,^b^	taVNS probably increases motor function
Mental health	Randomised trials	Not serious	Not serious	Serious^c^	Serious^d^	SMD 0.84 higher (0.49 higher to 1.19 higher)	⨁⨁⨁◯ Moderate^c^^,^^d^	taVNS probably increases mental health
ADL	Randomised trials	Serious	Serious^e^	Serious^c^	Not serious	SMD 0.94 higher (0.72 higher to 1.17 higher)	⨁⨁◯◯ Low^c^^,^^e^	taVNS may increases ADL
Neuroelectrophysiological	Randomised trials	Serious	Very serious^f^	Serious ^c^	Serious ^g^	SMD 1.6 higher (0.7 higher to 2.51 higher)	⨁◯◯◯ Very low^c^^,^^f^^,^^g^	The effect of taVNS on neuroelectrophysiological is very uncertain

CI, confidence interval; SMD, standardised mean difference.

a Downgraded by one level due to *I*^2^ = 63% without a plausible explanation through subgroup analysis.

b Downgraded by one level because 2 out of the 8 included studies used different control interventions compared to the other 6 studies.

c Downgraded by one level due to inconsistency in control interventions across the included studies.

d Downgraded by one level due to the total number of events being less than 150.

e Downgraded by one level because the *I*^2^ = 62% and could not be explained by subgroup analyses.

f Downgraded by two levels due to an *I*^2^ = 92% that could not be explained by subgroup analyses, with some studies even reporting effects in opposite directions.

g Downgraded by one level due to wide confidence intervals.

### Main efficacy outcome analyses

**Figure 3:**
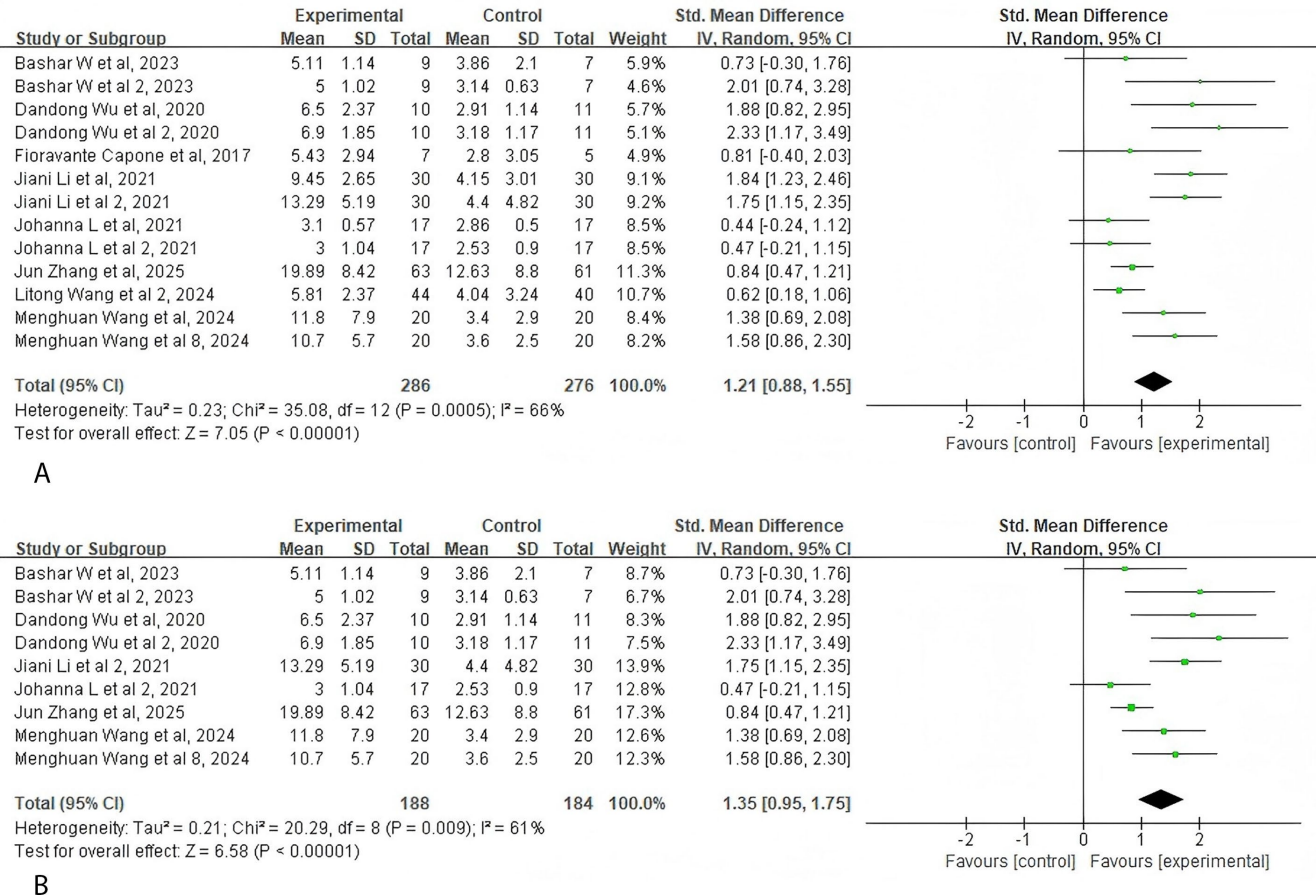
Forest plot of the effect of taVNS on motor function. **(A)** Motor function. **(B)** Upper limb motor function.

A total of 8 studies (286 participants) evaluated motor function in patients following intervention. The studies employed the FMA, ARAT, and WMFT scales. The pooled analysis of data from these 286 participants revealed that patients in the taVNS group demonstrated higher motor function scores than the participants in the control group (SMD = 1.21, 95% CI: 0.88–1.55, *p* < 0.001). The quality of evidence is moderate (Figure 3A).

The pooled meta-analysis of inter-study data indicated that the taVNS group demonstrated superior upper limb motor function scores compared with the control group post-intervention (SMD = 1.35, 95% CI: 0.95–1.75, *p* < 0.001) (Figure 3B).

When evaluating upper limb functional efficacy post-intervention using the FMA-UE scale (SMD = 1.57, 95% CI: 0.99–2.15, *p* < 0.001) and the WMFT scale (SMD = 0.96, 95% CI: 0.14–1.78, *p* = 0.02), the taVNS group demonstrated superior outcomes compared with the control group. However, when assessed using the FMA-LE scale (SMD = 1.21, 95% CI: 0.02–2.41, *p* = 0.05), no significant differences in efficacy were observed between groups.

### Additional efficacy outcome analyses

**Figure 4:**
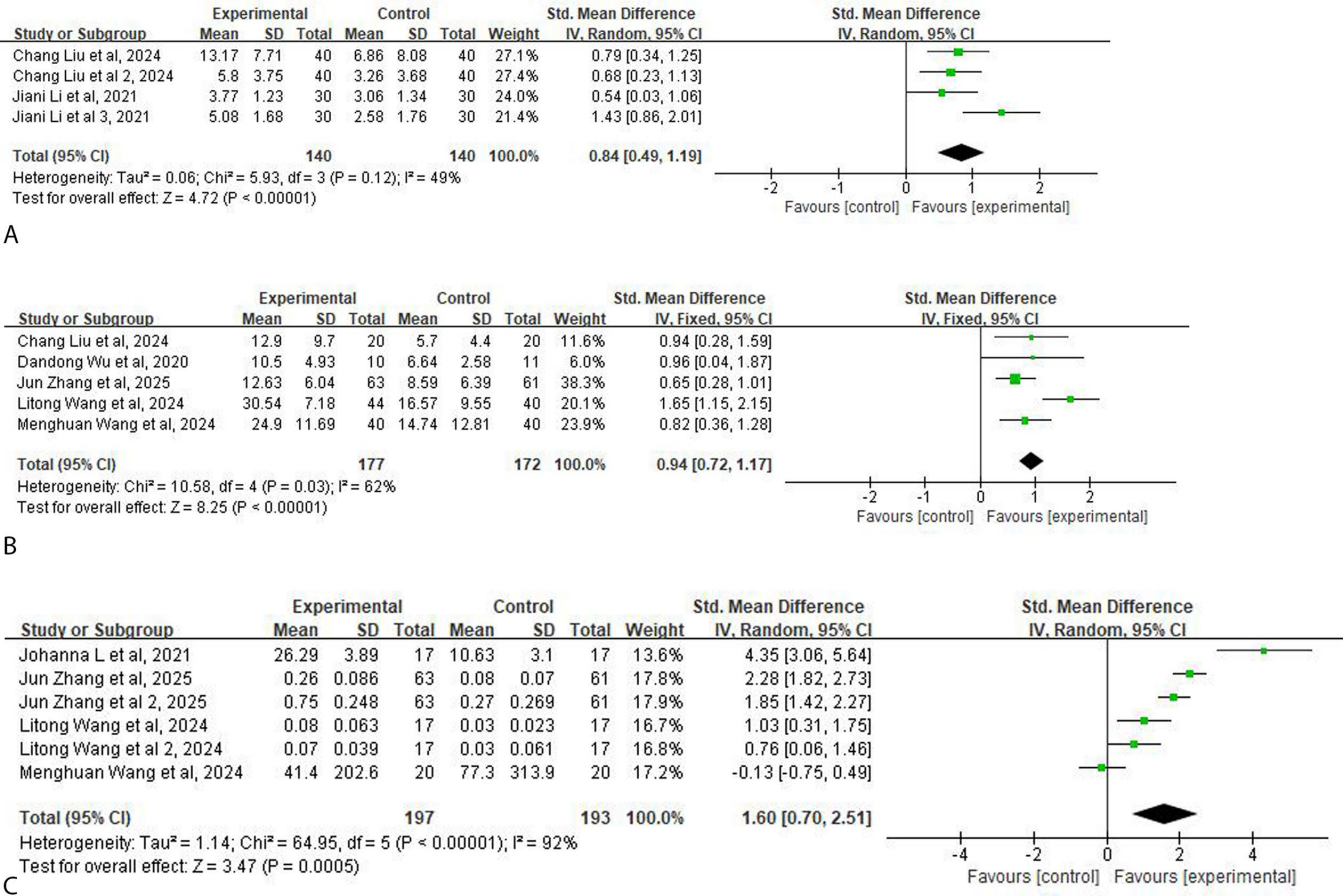
Forest plot of the effect of taVNS on additional outcomes. **(A)** Mental health. **(B)** ADL. **(C)** Neuroelectrophysiology.

Two studies (140 participants) assessed psychological functioning, using 4 scales: HADS-A, HADS-D, HADM-17, and SDS. The meta-analysis results revealed that the post-intervention psychological function scores were significantly lower in the taVNS group than in the control group (SMD = 0.84, 95% CI: 1.19–0.49, *p* < 0.001), indicating superior improvement in psychological function among patients with taVNS compared with controls. The quality of evidence is moderate (Figure 4A).

Five studies (349 participants) assessed ADL outcomes using the BI, MBI, and FIM scales. The pooled analysis revealed a statistically significant difference in scale scores between the taVNS and control groups post-intervention (SMD = 0.94, 95% CI: 0.72–1.17, *p* < 0.001). The quality of evidence is low (Figure 4B).

In terms of neurophysiology, four studies selected two indicators for assessment: EMG and MEP. The meta-analysis revealed that the taVNS group demonstrated significantly greater neurophysiological improvement compared with the control group after intervention (SMD = 1.60, 95% CI: 0.70–2.51, *p* = 0.0005) The quality of evidence is very low (Figure 4C).

### Subgroup analyses

Subgroup analyses were conducted for the combined analysis of primary and secondary efficacy outcomes following taVNS intervention (based on pre-intervention stroke stage, conventional rehabilitation treatment parameters and taVNS stimulation parameters) (Table 3).

**Table 3:** Effectiveness of subgroup analysis in terms of patient characteristics and taVNS parameters.

Subgroup	Category	Studies	Experimental group, *n*	Control group, *n*	SMD (95% CI)	*p*
Phase of stroke onset	Acute/subacute	3	327	330	1.05 (0.56–1.53)	<0.001
	Subacute	4	592	571	1.11 (0.81–1.41)	
	Chronic	3	59	53	0.72 (0.26–1.18)	
taVNS frequency (Hz)	20	4	307	308	1.04 (0.59–1.49)	<0.001
	25	4	385	368	1.02 (0.72–1.33)	
	30	1	34	34	0.45 (−0.03 to 0.94)	
taVNS pulse width (ms)	200	1	252	244	1.39 (0.64–2.14)	<0.001
	300	6	568	553	0.68 (0.19–1.16)	
	500	3	175	174	0.83 (0.08–1.59)	
Total intervention course	≤10	2	58	56	1.44 (−0.01 to 2.88)	<0.001
	11–20	5	508	491	1.02 (0.71–1.34)	
	>20	3	429	424	1.23 (0.79–1.66)	
Weekly frequency of taVNS (sessions)	≤4	2	69	65	1.51 (0.31–2.71)	<0.001
	>4	8	886	866	1.09 (0.82–1.35)	
Duration of per session of conventional rehabilitation (h)	≥1	3	75	71	0.38 (0.05–0.72)	<0.001
	<1	5	342	328	0.78 (−0.06 to 1.61)	
Total number of sessions of conventional rehabilitation (sessions)	≤15	4	72	58	0.49 (0.02–0.95)	<0.001
	>15	4	355	341	0.68 (−0.16 to 1.51)	

The subgroup analysis revealed that taVNS yielded improved outcomes for stroke patients at any stage. However, the greatest efficacy was observed in patients during the subacute phase (SMD = 1.11, 95% CI: 0.81–1.41, *p* < 0.001). Regarding taVNS frequency, 20 Hz (SMD = 1.04, 95% CI: 0.59–1.49, *p* < 0.001) outperformed both 25 Hz (SMD = 1.02, 95% CI: 0.72–1.33, *p* < 0.001) and 30 Hz (SMD = 0.45, 95% CI: −0.03 to 0.94, *p* = 0.06). For taVNS total sessions, >10 sessions (SMD = 0.79, 95% CI: 0.08–1.50, *p* = 0.03) showed superiority over ≤10 sessions (SMD = 1.44, 95% CI: −0.01 to 2.88, *p* = 0.05); No statistically significant differences were observed between groups in terms of taVNS pulse width or taVNS weekly frequency. In terms of the duration of a single session of conventional rehabilitation, sessions lasting ≥1 h [SMD = 0.38, 95% CI (0.05, 0.72), *p* = 0.02] were superior to those lasting <1 h [SMD = 0.78, 95% CI (−0.06, 1.61), *p* = 0.07]. Regarding the total number of sessions of conventional rehabilitation, ≤15 sessions [SMD = 0.49, 95% CI (0.02, 0.95), *p* = 0.04] were better than >15 sessions [SMD = 0.68, 95% CI (−0.16, 1.51), *p* = 0.11].

## Discussion

The conclusions of this meta-analysis of data from 10 RCTs investigating the effects of taVNS on motor function, mental health, and ADL in patients with stroke were as follows. Eight motor function studies, two mental health studies, and five ADL studies confirmed that taVNS improved motor function, mental health, activities of daily living, and neurophysiological parameters in patients with stroke, with superior effects compared with those in the control group.

In the process of including the literature for this meta-analysis, some original studies contained multiple intervention groups that met the inclusion and exclusion criteria. To avoid the issue of non-independence of effect sizes caused by including multiple groups from the same literature, we took the following measures: Firstly, for all subgroup analyses, we prioritized the principle of “only including one group from each article”, and selected the intervention group that best met the inclusion and exclusion criteria for combination. Secondly, for a few studies that indeed needed to include multiple groups, two researchers independently verified whether there was any overlap in the sources of the subjects in each group, and only included them after confirming no overlap. Finally, we excluded a subgroup analysis in the original text that split 5 articles into 12 effect sizes. This part had a relatively high risk of non-independence due to excessive splitting.

Multiple studies have shown that the combined application of taVNS with various rehabilitation methods has a better therapeutic effect than taVNS alone. This is mainly based on its complementary mechanism for the functional recovery of patients. For instance, Wang et al. (19) combined taVNS with tDCS in post-stroke hemiplegic patients to observe the effects of the two on the lower limb movement and walking function of stroke patients. That is, taVNS and tDCS can promote neural plasticity at different levels: tDCS pre-activates the motor cortex, enhancing the response to subsequent stimuli; taVNS creates a neuroelectrophysiological environment conducive to neural plasticity. In another study (20), taVNS was conducted simultaneously with task-oriented training, using the “stimulus-task pairing” neural regulation strategy, which not only enhanced cortical plasticity but also guided neural plasticity towards a functional direction. However, among the 10 included studies in this research, only 4 combined taVNS with other rehabilitation methods, which might be due to the small number of included studies. Due to the small sample size, the results of the subgroup analysis may be biased.

The mechanism of taVNS varies when applied at different stages of stroke recovery. In the early stage after stroke, the neural circuits of patients are mainly characterized by edema or inflammatory responses, with blocked neural conduction and generally low cortical excitability. At this time, the application of taVNS can take advantage of its neuroprotective effects in the early stage after stroke, such as anti-infection and protection of the blood–brain barrier, regulation of neuroinflammation, and maintenance of cortical excitability. In the subacute stage, cortical function begins to be remodeled. At this stage, the application of taVNS can enhance the activation of task-related cortices, achieving maximum functional recovery. In the chronic stage, although spontaneous recovery tends to plateau, the observed functional improvements in previous trials suggest that at this time, taVNS does not directly act on the lesion but rather through a mechanism independent of neuroprotection, namely, by increasing the levels of brain-derived neurotrophic factor (BDNF) and norepinephrine, and other neurotransmitters related to neural plasticity and recovery after brain injury.

This study included multiple RCTs and found that taVNS promoted recovery of motor function after stroke. This result was consistent with the findings from previous meta-analyses (21). This meta-analysis involved a subgroup analysis of the effects of various types of NIBS on functional recovery in patients with stroke by including 87 RCTs. The results indicated that taVNS significantly outperformed other NIBS modalities in improving post-stroke motor function. This superiority might have stemmed from the ability of taVNS to elevate the levels of neurotrophic factors and neurotransmitters such as norepinephrine, which are closely associated with neural plasticity and functional recovery following brain injury. For example, relevant studies indicated that stimulating the taVNS activated the locus coeruleus-norepinephrine (LC-NE) release system, inducing sustained norepinephrine (NE) activity (22). This increased cortical excitability and plasticity, enhancing motor learning capacity (23). Additionally, taVNS specifically influenced inhibitory activity in the motor cortex. It modulated the balance between excitatory and inhibitory neurotransmission by selectively enhancing the function of inhibitory interneurons, thereby promoting functional recovery in patients with stroke (24). Although taVNS demonstrates significant potential in post-stroke motor recovery, both the experimental and control groups in a previous study exhibited marked improvements in motor function after taVNS application, with no significant differences between groups. This might be attributed to the study’s failure to synchronize taVNS with other rehabilitation therapies over time, thereby preventing the coupling effects between taVNS and rehabilitation treatments from manifesting (8).

Approximately 20–60% of stroke survivors experience mental health issues such as post-stroke depression (25). This has a series of serious consequences, including reduced efficacy of physical rehabilitation, prolonged hospital stays, and diminished self-efficacy. However, only two trials examined the effects of taVNS on post-stroke mental health issues, concluding that taVNS effectively improved these conditions. This finding was inconsistent with the results of a meta-analysis conducted by Gao et al. This discrepancy might have stemmed from their meta-analysis, which combined results from studies using different types of VNS devices (10).

Five RCTs evaluated the impact of taVNS on ADLs in patients with stroke. All assessment tools used (MBI, BI, and FIM) indicated that taVNS improved post-stroke ADLs. The meta-analysis revealed no statistically significant difference in ADL improvement between the taVNS and control groups. This finding aligned with the results of previous meta-analyses (21). Another meta-analysis combining three RCTs found no statistically significant difference in ADL improvement with taVNS (10). However, the conclusions of the aforementioned meta-analysis were derived from combining three studies that employed different types of VNS devices and multiple assessment scales.

Beyond exercise and mental health, taVNS may also promote the recovery of swallowing function after stroke. Wang et al. (26) applied taVNS to the auricular branches of the vagus nerve on both sides of the ears in patients with post-stroke dysphagia. The swallowing function significantly improved after a 3-week intervention compared with that in the control group. Previous studies have confirmed that taVNS activates the nucleus tractus solitarius and locus coeruleus (LC)—key components of the swallowing pattern generator (27)—promoting laryngeal sensory recovery and pharyngeal muscle regulation. This, in turn, improves cough reflex and soft palate function (28).

### Time-dependent

Neural structural changes and regeneration are key to functional recovery after stroke. Rehabilitation training enhances blood flow perfusion around the infarct area, promotes neurogenesis, neuronal differentiation, and survival following brain injury, inhibits neuronal apoptosis, prunes dendritic spines, and modulates synaptic plasticity (29).

Multiple studies indicate that VNS combined with rehabilitation therapy enhances cortical reorganization capacity, doubling the number of synaptic connections within the corticospinal tract network regulating motor function (30), thereby further promoting functional recovery after stroke (31).

However, the timing of combining taVNS with rehabilitation training is crucial. When taVNS is administered after rehabilitation training, the synergistic effect between the two does not manifest (32). Khodaparast et al. (33) divided 29 rats with chronic ischemic stroke into 3 groups: a VNS-synchronized rehabilitation training group, a delayed group receiving VNS 2 h after daily rehabilitation training, and a rehabilitation training alone group. The results showed that the forelimb muscle strength recovery rate reached 85.9% ± 6.1% in the paired VNS group, 42.1% ± 8.0% in the delayed VNS group, and only 47.2% ± 13.4% in the rehabilitation-only group. These findings indicated that VNS-synchronized rehabilitation training significantly improved forelimb functional recovery compared with equivalent rehabilitation training without VNS. Delayed VNS administered 2 h after daily rehabilitation failed to promote functional recovery, indicating that VNS must be temporally synchronized with rehabilitation to be effective. This finding confirmed that the synergistic effect of VNS and rehabilitation depended on neuroplasticity—a time-dependent physiological phenomenon.

Some studies have further expanded upon this theory by implementing taVNS prior to rehabilitation training to observe functional recovery in patients with stroke. Capone et al. (34) enrolled 12 patients with chronic stroke and randomly assigned them to 2 groups: immediate robotic rehabilitation training following taVNS versus sham stimulation plus robotic rehabilitation training. The results showed that the FMA score in the true stimulation group increased from 22.3 to 27.7, representing a 9% improvement rate; the FMA score in the sham stimulation group increased from 32.6 to 35.4, representing a 4% improvement rate. This study demonstrated that implementing VNS prior to rehabilitation also improved motor function in patients with stroke.

This study classified the stimulation timing into “synchronous stimulation,” “pre-rehabilitation stimulation,” and “post-rehabilitation stimulation” based on the intervention protocols described in the included original studies. However, even within the category of “synchronous stimulation,” there was significant heterogeneity among different studies regarding the specific duration of each stimulation session (such as throughout the entire rehabilitation training or only a few minutes before the training) and the specific application points of stimulation within a single session (such as immediately before the training or intermittently during the training). This heterogeneity may be an important moderating variable affecting the overall effect size. However, due to the insufficient reporting of intervention protocol details in the original data of the current included studies and the limited number of included studies, quantitative subgroup analyses based on these parameters could not be conducted in this analysis. Future studies should standardize the reporting of intervention protocol parameters to more precisely parse the dose-effect relationship and time window effect of taVNS.

Experimentation further defined the timing of taVNS relative to other rehabilitation exercises (8), thereby developing a closed-loop neuromodulation system. This system integrated EMG signals with taVNS to construct a closed-loop framework for motor rehabilitation. The system detected movement via surface EMG sensors placed on the anterior and medial deltoid muscles. Upon detecting muscle activity whose resulting EMG signal exceeded the stimulation threshold, the neurostimulator delivered low-intensity electrical currents to two specific targets in the participant’s ears within 100 ms. This system aimed to synchronize taVNS with repetitive movements during post-stroke motor rehabilitation, thereby achieving millisecond-level precision pairing (<100 ms) between taVNS and rehabilitation training. Subsequently, the system was applied in a prospective, randomized, double-blind trial involving 20 participants with chronic unilateral upper-limb motor dysfunction. The trial validated whether synchronizing stimulation with training movements improved efficacy compared with nonintelligent, nonpaired taVNS protocols. The results indicated that movement-synchronized stimulation might yield benefits by optimizing stimulation timing: Even with significantly reduced total stimulation duration in the MAAVNS group, its effect size was twice that in the nonpaired taVNS group. This suggested the protocol might establish a more efficient therapeutic model.

Although the above-mentioned closed-loop neuromodulation system has demonstrated the great potential of “precise pairing,” the majority of the trials included in this study generally ignored this key principle. Current research mostly adopts an open-loop design, that is, continuous or regular stimulation at fixed time intervals during rehabilitation training, rather than closed-loop triggering based on the real-time performance of the patient. This may be due to the lack of real-time physiological signal acquisition and analysis modules in traditional stimulation devices, making it difficult to achieve low-latency closed-loop triggering. The confounding effect of this open-loop stimulation may be an important reason for the high heterogeneity and low effect size in current taVNS research. Therefore, future taVNS research urgently needs to shift to a closed-loop paradigm. This on-demand precise stimulation can not only avoid unnecessary neural adaptation or tolerance, but also precisely target the plasticity window. In future meta-analyses, distinguishing between “closed-loop precise stimulation” and “open-loop continuous stimulation” will be a key step to reduce heterogeneity and reveal the true effect size.

### taVNS parameters

The consensus on the “optimal” stimulation parameters for taVNS is lacking. The subgroup analysis of taVNS stimulation parameters in this study offers novel insights into key factors influencing the efficacy of taVNS following stroke. Lower-frequency taVNS (20 Hz) may yield better outcomes in patients with stroke compared with higher frequencies (>20 Hz). This finding aligns with the results of the meta-analysis by Wang et al. (35) on VNS for post-stroke upper limb dysfunction. The study included 10 research projects. The subgroup analysis of VNS frequencies revealed that low-frequency stimulation (20 Hz) might demonstrate superior therapeutic efficacy for post-stroke motor dysfunction compared with high-frequency stimulation (25 or 30 Hz). Collectively, this evidence suggests that stimulation at a frequency of 20 Hz may yield superior therapeutic outcomes.

The current intensity of taVNS is another key parameter influencing its therapeutic efficacy (36), and it interacts with the selection of pulse width. Increased current intensity promotes the release of neurotransmitters such as norepinephrine and elevates the firing frequency of locus coeruleus neurons (37). Therefore, nearly all taVNS studies employ current intensities exceeding the perception threshold. When current intensity is reduced to 0.2 mA, the promotion of neural plasticity by VNS disappears; however, this effect can be restored by appropriately extending the pulse width at low intensities. This suggests that shorter pulse widths may exert a “permissive effect” on current intensity, thereby enabling a broader range of current intensities to drive neural plasticity (38). In other words, pulse width can partially compensate for limitations in current intensity, but combining high intensity with wide pulses can induce tolerance issues. In summary, future studies should systematically investigate the interaction between current intensity and pulse width to identify the optimal parameter combination for both.

### Neurophysiological measures

This study found that although the overall effect of taVNS on neurophysiological indicators was significant, the high heterogeneity indicated that this effect was highly unstable across different studies. The source of this heterogeneity is likely directly related to the differences in stimulation parameters and timing among the studies.

Firstly, in terms of stimulation parameters, there were significant differences in the intensity, frequency, pulse width, and electrode placement among the studies. This lack of uniformity in dosage may lead to significant differences in the magnitude of neurophysiological responses. Secondly, there is the issue of the synchronization between stimulation and movement. Previous studies have confirmed that stimulation must be given within hundreds of milliseconds of a successful movement to specifically enhance motor evoked potentials. However, the studies included in this meta-analysis mostly used open-loop continuous stimulation, with only a few using closed-loop precise stimulation. The former provides a non-specific stimulation intervention, while the latter can precisely target the plasticity window. Combining these two distinct stimulation modes for analysis is undoubtedly the direct cause of the high heterogeneity.

In conclusion, the existing evidence suggests that taVNS may have neurophysiological effects on the central motor conduction pathway and muscle excitability. However, due to the ‘very low’ quality of the evidence and the high heterogeneity among studies, this conclusion is highly uncertain. Future research should: establish and report standardized stimulation parameters; prioritize the use of closed-loop (task-matched) stimulation paradigms to achieve precise pairing with neural activity; and report “continuous stimulation” and “precise stimulation” separately in the analysis. Only after these methodological issues are resolved can reliable judgments be made about the effects of taVNS on regulating neuroelectrophysiology.

## Limitations

This study had certain limitations. First, in the initial literature search, a significant number of studies were found to be registered only in clinical trial registries without subsequent publication of their results, indicating substantial publication bias. Second, among the published studies, the findings were predominantly positive, whereas negative results might have been withheld. This limited the sample size of included studies, potentially leading to an overestimation of the actual efficacy of taVNS in post-stroke recovery within the meta-analysis. Therefore, researchers are encouraged to report all outcomes truthfully in future studies, regardless of results. Clearer associations between taVNS rehabilitation efficacy and patient demographics, disease characteristics, and optimal treatment parameters remain elusive. Moreover, there is no standardized reporting of the parameters of the intervention program in the existing studies, which makes it impossible to quantitatively analyze the relationship between treatment parameters and efficacy. Future studies should standardize the reporting of intervention program parameters in order to more precisely resolve the dose–response relationship and time window effects of taVNS, so as to develop interventions more effectively.

## Conclusion

The results of this meta-analysis indicated that taVNS improved motor function, mental health, ADL, and neurophysiological indicators in patients with stroke. The current intensity, pulse width, and total number of treatment sessions of taVNS are key factors influencing its therapeutic efficacy.

## Data Availability

The original contributions presented in the study are included in the article/supplementary material, further inquiries can be directed to the corresponding author.

## Footnotes

1
https://gdt.gradepro.org/app/
