# Correction: Effectiveness of transcutaneous auricular vagus nerve stimulation in stroke rehabilitation: a systematic review and meta-analysis of randomized clinical trials

**DOI:** 10.3389/fneur.2026.1881102

**Published:** 2026-05-29

**Authors:** Shuaijing Wan, Xiaolu Liu, Wenjing Jiang, Zesen Li, Zhexuan Yan, Yu Yin, Weibo Li

**Affiliations:** 1 College of Nursing and Rehabilitation, North China University of Science and Technology, Tangshan, China; 2 Department of Rehabilitation, Hebei General Hospital, Shijiazhuang, China; 3 Hebei Provincial Key Laboratory of Cerebral Networks and Cognitive Disorders, Shijiazhuang, China; 4 Graduate School of Hebei Medical University, Shijiazhuang, China; 5 Physical Education College, Hebei Normal University, Shijiazhuang, China; 6 Department of Gastrointestinal Surgery, The Second hospital of Hebei Medical University, Shijiazhuang, China

**Keywords:** meta-analysis, motor, stroke, taVNS, transcutaneous auricular vagus nerve stimulation

In the published article, the order of authors in the author list was erroneously given as:

Shuaijing Wan^1, 2^, Xiaolu Liu^2, 3^, Wenjing Jiang^4^, Zesen Li^4^, Zhexuan Yan^5^, Weibo Li^6^^*^, and Yu Yin^1, 2,3^^*^

The correct author list reads:

Shuaijing Wan^1, 2^, Xiaolu Liu^2, 3^, Wenjing Jiang^4^, Zesen Li^4^, Zhexuan Yan^5^, Yu Yin^1, 2,3^^*^ and Weibo Li^6^^*^

The original version of this article has been updated.

